# Pituitary hyperplasia secondary to primary hypothyroidism

**DOI:** 10.1002/ccr3.2863

**Published:** 2020-04-16

**Authors:** Beatriz Martinez Quintero, Cynthia Yazbeck

**Affiliations:** ^1^ Department of Internal Medicine. Division of Endocrinology, Diabetes and Metabolism Virginia Commonwealth University Richmond VA USA; ^2^ Department of Internal Medicine. Division of Endocrinology, Diabetes and Metabolism Hunter Holmes McGuire VA Medical Center Richmond VA USA

**Keywords:** hypothyroidism, pituitary gland, pituitary hyperplasia, primary hypothyroidism

## Abstract

Severe primary hypothyroidism should be considered in the differential diagnosis of pituitary enlargement. Thyroid hormone replacement therapy should lead to regression of pituitary hyperplasia.

## Graphical abstract

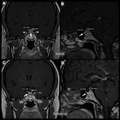

A 53‐year‐old woman with migraine headaches and primary hypothyroidism on replacement therapy with levothyroxine underwent magnetic resonance imaging (MRI) of the brain for evaluation of worsening headaches that incidentally found an enlarged pituitary gland. MRI of the pituitary demonstrated an enlarged pituitary gland measuring 1.1 × 0.8 × 0.8 cm, without suprasellar extension, involvement of the cavernous sinuses, or mass effect on the optic chiasm (Figure 1, panel A, B). On further assessment of her symptoms, she also reported the gradual onset of fatigue, weight gain, constipation, and cold intolerance after starting to take calcium supplements in conjunction with levothyroxine. Laboratory evaluation revealed thyroid‐stimulating hormone (TSH) of 247 mIU/mL with free thyroxine (T4) of 0.3 ng/dL. Her levothyroxine dose was increased, and she was advised to separate it at least 4 hours from calcium supplements. Six months later, her symptoms improved and her TSH nearly normalized to 4.22 mIU/mL and her free T4 normalized to 0.9 ng/dL. A follow‐up MRI was obtained which showed that the size of the pituitary gland had dramatically decreased (Figure 1, panel C, D). She was diagnosed with uncontrolled primary hypothyroidism leading to pituitary hyperplasia.

**Figure 1:**
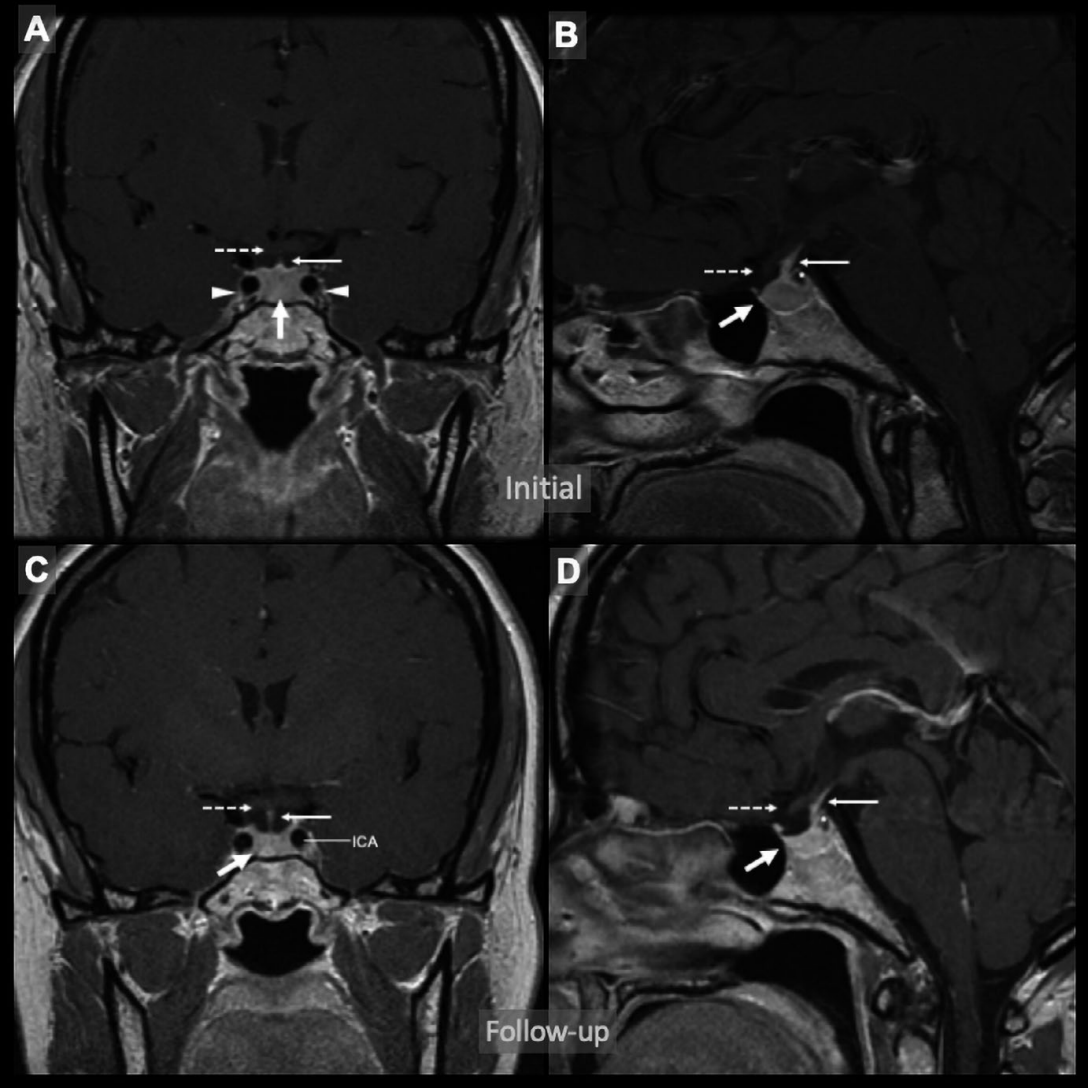
A, Coronal view and (B) sagittal view of the initial contrast‐enhanced magnetic resonance imaging (MRI) of the pituitary gland showing an enlarged and homogeneously enhanced pituitary (thick arrows) measuring 1.1 × 0.8 × 0.8 cm, consistent with pituitary hyperplasia; as well as a cystic focus (asterisk) at the superior portion of the neurohypophysis extending toward the posterior margin of the infundibulum (solid thin arrow) without displacing it. No mass effect on the optic chiasm (dashed thin arrows), no suprasellar extension, or involvement of the cavernous sinuses (arrowheads) identified. C, Coronal view and (D) sagittal view of the follow‐up MRI of the pituitary gland showing a normal‐sized pituitary (thick arrows) along with a stable subcentimeter cystic focus (asterisk) at the superior portion of the neurohypophysis likely representing a pars intermedia or Rathke's cleft cyst. No mass effect on the optic chiasm (dashed thin arrows) or extension into adjacent structures identified. Pituitary infundibulum is indicated with solid thin arrows. ICA, internal carotid artery

Severe primary hypothyroidism should be considered in the differential diagnosis of pituitary enlargement as the lack of negative feedback from T4 leads to elevated levels of thyrotropin‐releasing hormone causing pituitary thyrotrophs stimulation and hyperplasia with consequent pituitary enlargement. Thyroid hormone replacement should lead to regression of pituitary hyperplasia.^1^

## CONFLICT OF INTEREST

None declared.

## AUTHOR CONTRIBUTIONS

All authors have made a substantial contribution to the preparation of this manuscript. BMQ: wrote the initial draft. CY: contributed with the acquisition and interpretation of the images for this manuscript. All authors participated in the analysis and interpretation of the data, and critical revision of the manuscript for important intellectual content. All authors reviewed the final version of the manuscript and approved its submission.
